# The Usefulness of 55° Wide-Field Spectral-Domain Optical Coherence Tomography in Monitoring the Features of Peripheral Subretinal Fluid Remnants after Rhegmatogenous Retinal Detachment Surgery

**DOI:** 10.3390/diagnostics14131385

**Published:** 2024-06-29

**Authors:** Valentina Carta, Filippo Lixi, Pasquale Loiudice, Francesca Frongia, Filippo Tatti, Chiara Delpiano, Pierluca Cremonesi, Enrico Peiretti

**Affiliations:** 1 Department of Surgical Sciences, Eye Clinic, University of Cagliari, 09124 Cagliari, Italy; vale.carta94@gmail.com (V.C.); f.lixi0106@gmail.com (F.L.); fr.frongia@gmail.com (F.F.); filippotatti@gmail.com (F.T.); chiara.delpi@gmail.com (C.D.); pierluca.cremonesi@gmail.com (P.C.); 2 Ophthalmology Complex Operative Unit, “F. Lotti” Hospital, 56025 Pontedera, Italy; ldcpasquale@gmail.com

**Keywords:** rhegmatogenous retinal detachment, subretinal fluid, spectral-domain OCT, wide-field OCT

## Abstract

Background: This study aimed to assess the effectiveness of 55° wide-field (WF) spectral-domain (SD) optical coherence tomography (OCT) for detecting peripheral subretinal fluid (SRF) after surgery for rhegmatogenous retinal detachment (RRD). Methods: In this retrospective observational study, the retinal periphery was examined to evaluate the possible presence of persistent SRF after surgery. OCT scans were acquired in infrared mode to use any peripheral vessel as a landmark for better repeatability in monitoring fluid remnants. Results: A total of 80 patients (10% with high myopia) were examined using 55° WF SD OCT after successful pars plana vitrectomy (83.8%) or scleral buckling (16.3%) for RRD. A total of 18 patients (22.5%), 16 of whom underwent pars plana vitrectomy and 2 who underwent scleral buckling, showed SRF at the OCT examination during the follow-up. Potential risk factors associated with SRF persistence were analyzed, revealing a significative association with young age (*p* = 0.009). After a follow-up period of 7.05 ± 2.44 months (ranging from 3 to 12 months), a complete resorption in all patients (100%) within 12 months was observed. Best-corrected visual acuity significantly improved in both groups over time. Conclusion: Using 55° WF SD-OCT successfully assessed the course of SRF reabsorption, offering a viable alternative for all those realities where technologies such as ultra-wide-field (UWF) OCT are not available.

## 1. Introduction

Shallow subretinal fluid (SRF) may persist after surgery for rhegmatogenous retinal detachment (RRD), even when the retina appears fully attached on ophthalmoscopic examination [1]. SRF may be defined as the presence of shallow, concave fluid under or around the fovea, or as peripheric or focal accumulations of fluid corresponding to SRF blebs [1]. Machemer was the first who described small gatherings of SRF after resolution of experimental retinal detachment in owl monkeys in 1983 [2].

After surgery for RRD, the persistence of SRF has been observed in more than 50% of patients who underwent scleral buckling (SB) and in up to 15% of those who underwent pars plana vitrectomy (PPV) [3,4,5]. The increased occurrence of persistent SRF following SB can be attributed to the fact that in the absence of PPV, not all the fluid is completely drained during the surgery, leading to the development of small SRF accumulations after the surgery [6].

The advent of optical coherence tomography (OCT) has dramatically changed the evaluation of patients with macular and vitreoretinal diseases. For identifying and monitoring SRF, which may elude detection during conventional clinical exams, OCT represents a valuable, noninvasive tool. As illustrated by Cereda et al., OCT imaging is helpful for evaluating SRF even in cases of rhegmatogenous retinal detachment (RRD), allowing for the identification, measurement, and study of RRD characteristics, thereby improving management and aiding in treatment decisions [7]. Furthermore, shallow SRF, characterized by a height of less than 300 μm, is often not detectable during routine funduscopic examination and requires the use of OCT [1]. Its evaluation is a critical moment during follow-up, as it is of essential importance to investigate any dehiscence of the causative retinal break or identify any small, misdiagnosed holes and therefore evaluate the need for a new surgery.

The standard field of view in OCT systems is 30°, primarily suited for examining conditions affecting the macula. However, advances in imaging technology have led to a new era, introducing wide-field (WF)- and ultra-wide-field (UWF)-OCT systems capable of capturing a 55° and a 200° field of view (FOV), respectively. UWF-OCT allows the identification of the far periphery of the retina up to the pars plana; however, its high cost limits its accessibility in certain healthcare departments [8]. Herein, our aim was to assess the effectiveness of a 55° wide-field (WF) Spectralis spectral-domain (SD)-OCT for detecting peripheral SRF during the follow-up of patients after successful surgery for RRD.

## 2. Materials and Methods

This was a retrospective observational study conducted at the University Hospital of Cagliari, Italy. This study was performed in accordance with the International Conference on Harmonization-Good Clinical Practice (ICH-GCP) guidelines and adhered to the tenets of the Declaration of Helsinki.

### 2.1. Patients’ Selection

The medical records of 80 consecutive patients who underwent naïve retinal detachment surgery from March 2022 to June 2023 were examined. Successful surgery was defined as complete retinal reattachment after a single surgery. In the series, 13 and 67 eyes (10% with high myopia) underwent SB and PPV, respectively, with a minimum postoperative follow-up period of 3 months. All patients were evaluated at 1 week, 1 month, and 3 months postoperatively. In cases of persistent SRF, follow-up visits were arranged monthly until complete absorption of the SRF.

Subjects with a history of ocular surgery (except cataract surgery), proliferative vitreoretinopathy grade C (Retina Society Terminology Committee Classification) [9] at presentation, recent intraocular infection, serous or tractional retinal detachment, previous ocular trauma, and all the retinal detachment that needed silicon oil tamponade were excluded.

### 2.2. Data Collection

The following data were collected whenever accessible from each patient’s medical file: age, gender, systemic conditions like hypertension and diabetes mellitus, past eye surgeries, duration of detachment, refractive error, best-corrected visual acuity (BCVA) at baseline, presurgery and postsurgery intraocular pressure, preoperative lens condition and any combined surgery conducted, extent of detachment, count and position of tears, existence and severity of underlying proliferative vitreoretinopathy, specific type of surgery administered, presence of shallow peripheral SRF after the surgery, additional interventions due to the SRF, postoperative BCVA, and total follow-up duration. High myopia was considered as a spherical equivalent of −6 D or higher.

### 2.3. Surgical Technique

The surgeries were performed by a single surgeon (E.P.)

Small gauge pars plana vitrectomy: A standard 3-port 25 G PPV with the use of 20% SF6 gas as the tamponade agent was performed under local anesthesia (parabulbar block using 5 cc lidocaine and 5 cc bupivacaine. Surgical procedures were performed using a Constellation^®^ Vitrectomy system (Alcon, Fort Worth, TX, USA) and BIOM wide-angle viewing system (Oculus Optikgeräte GmbH, Wetzlar, Germany). Core and shave vitrectomy was followed by separation of the posterior hyaloid membrane, if necessary. SRF was partially drained through the original breaks under air. Subsequently, cryotherapy or endolaser treatment of the retinal breaks was performed.

Scleral buckling: Patients with multiple superior tears underwent scleral buckling with a silicone tire (No. 287; MIRA, Hong Kong, China) or silicone band (No. 240; Microvision Inc., Redmond, WA, USA) of 6 mm in width and 1.25 mm in height for encircling SB. Conversely, patients with one single superior tear were treated with a silicone sponge measuring either 5 mm or 7.5 mm in width and 1.25 mm in height (No. 506 or 507; MIRA, Waltham, MA, USA) for the segmental SB. Drainage puncture was performed below the buckling with respect to the highest elevation of the retina, ideally away from the vorticose veins and in proximity to the upper or lower edge of the horizontal rectus muscles or underneath the vertical rectus muscles.

### 2.4. Optical Coherence Tomography Assessment

All individuals’ retinal periphery was examined with 55° wide-field SD-OCT (Spectralis OCT, Heidelberg Engineering, Heidelberg, Germany) scans over 360° to check any presence of persistent SRF. Horizontal and vertical line scans were performed across the area of the subretinal fluid in order to monitor its extent and its changes during follow-up. OCT scans were acquired in infrared mode (IR) in order to use any peripheral vessel as a landmark for better repeatability in monitoring of the fluid remnant. OCT scans were also acquired in the areas of breaks or defects previously treated with either cryo- or laser therapy in order to detect any continuity with the persistent SRF area and monitor its extent. A key element in image acquisition was to request the patient’s maximum cooperation in obtaining the most extensive excursion in each position of gaze so that most of the entire retina could be monitored until the far periphery. For each patient, serial follow-up 55° SD-OCT images were obtained periodically to assess the natural course of the peripheral SRF and to estimate the resorption time during the follow-up. Patients were routinely monitored until there was complete resolution of the persistent SRF.

### 2.5. Statistical Analysis

Statistical analysis was performed using the Statistical Package for Social Science software (IMB SPSS Statistics, version 25 for Windows). The distribution of variables was assessed with the Kolmogorov–Smirnov and Shapiro–Wilk tests. Visual acuity was converted to logMAR for statistical analysis. We assumed the following conversions: counting fingers (CFs) = 1.8 logMAR; perception of hand movement (HM) = 2.3 logMAR; light perception = 2.8 logMAR; no light perception = 3 logMAR. Analysis of variance for repeated measures was performed to compare the mean BCVA for the follow-up periods. Parametric and nonparametric tests were used to compare normally and non-normally distributed variables, respectively, between groups. Kaplan–Meier analysis was used to show the survival time of SRF after surgery. Univariate and multivariable Cox proportional hazards regression analyses were used to evaluate risk factors for duration of SRF. Risk factors with a *p* value < 0.2 in univariate analysis were included in the multivariable analysis. A *p* value of < 0.05 was considered statistically significant.

## 3. Results

This study included 80 eyes of 80 patients; 18 eyes (18 patients) out of 80 (22.5%) developed peripheral extrafoveal SRF. The mean follow-up period was 7.05 ± 2.44 months (range 3–12 months).

The demographic and clinical characteristics are displayed in Table 1.

Among the 18 subjects (11 men and 7 women), the mean age was 52 ± 15.2 (range 21–74) years, and the mean interval between diagnosis and surgery was 3.17 ± 1.38 days (range 1–6 days). The right eye was involved in nine cases; four subjects had high myopia, defined as a myopic error greater than 6 diopters.

A total of 16 patients underwent pars plana vitrectomy, 2 of whom did so with combined phacoemulsification. The remaining two cases received scleral buckling. Comparing the subjects who developed SRF and the subjects who did not, the two groups significantly differed in mean age, days between symptom onset and surgery, and lens status. 

**Figure 1:**
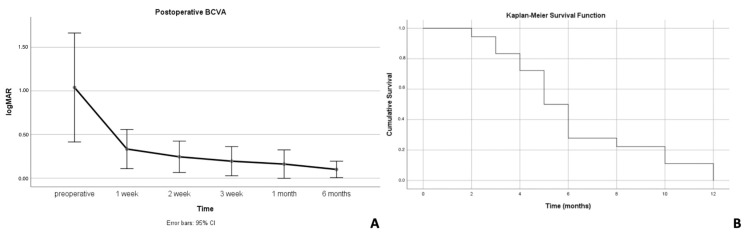
(**A**) Postoperative best-corrected visual acuity (BCVA) in subjects with subretinal fluid (SRF). (**B**) The survival probability of persistent subretinal fluid (SRF).

As shown in Table 1, younger age, duration between symptom’s onset and surgery and phakia showed a statistically significant association (*p* = 0.009; *p* = 0.019; *p* = 0) with the occurrence of extrafoveal SRF postoperatively. BCVA significantly improved in both groups over time. BCVA was logMAR 1.03 ± 0.81 at baseline and significantly increased to 0.1 ± 0.12 logMAR at the final follow-up (*p* = 0.005) (Figure 1A). BCVA, in the patients in whom SRF was not detected postoperatively, significantly increased from 1.2 ± 0.94 logMAR to 0.36 ± 0.43 logMAR at the final follow-up (*p* = 0.017). No difference was found comparing the final BCVA in both groups (*p* = 0.119, Mann–Whitney U test).

The mean SRF height at the 1-week postoperative visit was 226.9 ± 260.9 µm. The mean duration of SRF was 6.22 ± 3.00 months (range 2–12 months). SRF was already detectable 1 week after surgery in all cases. The prevalence of SRF after surgery was 94% at 2 months, 83% at 3 months, 72% at 4 months, 50% at 5 months, 27% at 6 months, 22% at 8 months, and 11% at 10 months. SRF was completely reabsorbed in all cases 12 months after surgery (Figure 1B).

Potential risk factors associated with SRF duration identified in the univariate analysis (*p* < 0.2) were preoperative BCVA, SRF height 1 week after surgery, macula status, high myopia, and type of surgery (Table 2). None of the aforementioned risk factors were significantly associated with SRF duration in the multivariate Cox regression analysis (Table 3).

## 4. Discussion

The presence of peripheral SRF is an extremely common finding after RRD surgery [1].

Although the pathogenesis is still not fully understood, it has been hypothesized that at the basis of this phenomenon, there may be an abnormal interaction between the retinal pigment epithelium (RPE) and photoreceptors. Normally, the apical villi of the RPE interdigitate, with the outer segments of the photoreceptors playing a crucial role in phagocytosis, disc renewal, and fluid reabsorption. Moreover, it is believed that these structures provide frictional resistance or an electrostatic force that prevents separation [10,11].

Within our cohort, 22.5% developed SRF, with localization of the collection in the inferior (55.55%), temporal (27.77%), nasal (11.11%), and superior sectors (5.55%). 

Younger age, phakia, high myopia, and long-standing inferior detachment have been suggested as possible preoperative risk factors for persistent SRF after surgery without, however, conclusive evidence [3,11,12,13,14].

In our study, we found, in accordance with the literature [11], an association between the persistence of SRF and different variables, such as younger age, lens status, days between symptom onset and surgery, and high myopia. This could be correlated with the fact that young patients are normally phakic; therefore, the lens inside the eye can make drainage maneuvers more difficult for the surgeon, especially in the periphery of the retina, and, in addition, they have less vitreous liquefaction and higher viscosity, which makes the absorption of SRF more difficult [15,16]. The lower incidence of SRF after PPV than that of SB also further supports the hypothesis that the vitreous state may be an influencing factor in this process [17,18]. We also speculate that, in our cohort of patients treated with PPV, there may have been a correlation between the higher percentage of SRF remnant and its partial intraoperative drainage under air without the use of heavy tamponade, such as perfluorocarbon liquid (PFCL), to displace the SRF. However, this hypothesis needs further data to be corroborated [6]. Moreover, 50% of the high-myopia patients showed persistence of peripheral SRF. Chen et al. [19] recently reported that retinal detachments in patients with high myopia were associated with a younger age at onset. It was reported that in patients with high myopia, a thinner and degenerated retinal pigment epithelium along with chorioretinal atrophy and a generally thinner choroid are associated with reduced pumping capabilities, causing greater difficulty in fluid resorption [20]. All these factors may contribute to the increased association with persistent SRF postoperatively. 

SD-OCT is a relatively recently developed, revolutionary, noninvasive, and user-friendly technology that provides high-resolution cross-sectional images of the retina, allowing detailed visualization of the retinal layers and subretinal structures. However, most available OCT devices are often limited by a relatively narrow FOV of approximately 30 degrees. Therefore, to address this gap in imaging capability, wide-field OCT technology (WF-OCT) with a FOV of around 55 degrees and ultra-wide-field OCT (UWF-OCT) with a FOV up to 200 degrees in a volumetric scan were developed to enable the evaluation of a larger area of the retina, which is crucial in RRD cases [21,22,23].

Furthermore, it is important to emphasize that the latest generation of UWF-OCT is an extremely expensive tool, and many centers do not have this technology, despite the high number of accesses for peripheral retinal pathologies, unlike WF-OCT, which is within the reach of most. Probably the only other way to examine the retinal periphery up to the ora serrata is by indirect ophthalmoscopy with 360° scleral indentation. However, due to a combination of lack of training and time during clinical examinations, this technique seems to be less performed at present, leaving more space for imaging techniques that are less invasive and less uncomfortable for the patient.

Using 55° SD-OCT, we were able to quantify the amount of SRF present and to track accurately its changes in level over time, aiding in the assessment of the effectiveness of treatment and the need for additional interventions.

**Figure 2:**
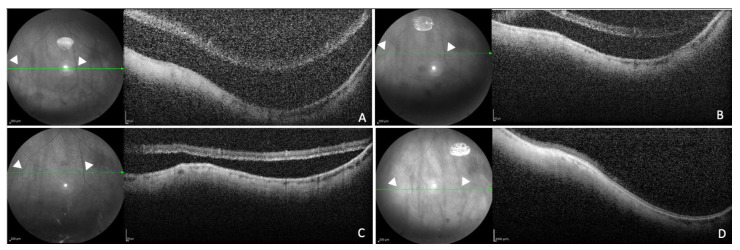
The 55° spectral-domain (SD) optical coherence tomography (OCT) infrared reflectance (IR)-guided scans displayed subretinal fluid (SRF) evolution after pars plana vitrectomy (PPV) 25 G. The same points marked by arrowheads on the IR images were used to monitor the evolution of fluid collections over time as precisely as possible. A 30-year-old patient with macula-off rhegmatogenous retinal detachment (RRD) 1 week (**A**), 1 month (**B**), 3 months (**C**) and 6 months (**D**) after surgery showing complete reabsorption of SRF.

Indeed, we monitored the progressive reduction in SRF in our study cohort until it was completely reabsorbed within 12 months of follow-up in all 18 patients (100%) (Figure 2).

In addition, by using the vessels and arterial or venous ramifications clearly visible in the infrared images as a landmark, it was possible to measure not only the fluid’s height but also to monitor its possible extension. In confirmation of this, our analysis allowed us to precociously highlight areas of dehiscence of intraoperatively cryotreated retinal breaks so as to eventually act promptly with a new surgery.

**Figure 3:**
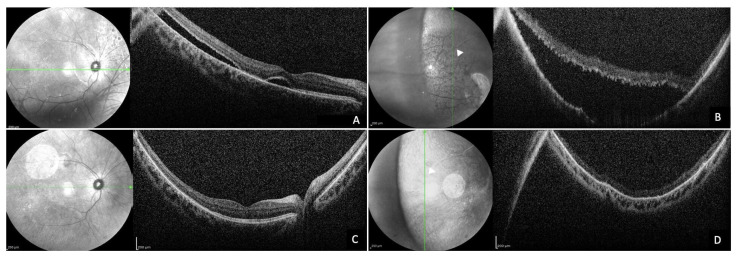
Infrared reflectance (IR) and 55° spectral-domain (SD) optical coherence tomography (OCT) images of a 21-year-old patient with macula-off rhegmatogenous retinal detachment (RRD), 2 weeks after SB showing SRF at the posterior pole (**A**) and the far temporal periphery (**B**), and at 10 months (**C**,**D**), unveiling a complete reabsorption of the fluid.

Previous studies [24,25] have shown that most peripheral SRF appeared about 1 month after surgery and disappeared within 12 months after surgery, and the present study is consistent with this time pattern, especially with regard to reabsorption times. Indeed, our cohort showed an average reabsorption of 6.22 ± 3.00 (range 2–12) months, where the subjects with the longest reabsorption time of the scleral buckles at 10 and 12 months were both young and highly myopic, confirming what has been described so far (Figure 3).

The limitations of our study include the small sample size and retrospective nature. Additionally, we have to consider that there was a bias regarding the number of patients who underwent PPV rather than SB. We encountered some limitations using our OCT system. The volumes were acquired by asking the patient to turn their gaze to extreme positions so as to reach the far periphery, and this requires a great deal of cooperation from the patient, which is not always easy to obtain. Additionally, in some cases, the fluid collection extended beyond this FOV, and, analyzing the extreme periphery, image distortions can occur, particularly in eyes with longer axial lengths.

## 5. Conclusions

To the best of our knowledge, this is the first report highlighting the efficacy and the usefulness of 55° SD-OCT for peripheral SRF monitoring after successful surgery for RRD. 

In our experience, WF-OCT has allowed us to obtain high-quality and clinically meaningful images of the peripheral retina, and it proved to be a key technique in the follow-up of all patients where fundus examination was not sufficient to evaluate shallow and very peripheral fluid variations, especially in myopic eyes.

This technique represents a viable alternative for all those realities where technologies such as UWF-OCT are not available, also helping in the decision-making process of whether or not to repeat surgery. 

## Figures and Tables

**Table 1:** Demographic and clinical characteristics.

Characteristics	Subretinal Fluid	No Subretinal Fluid	*p* Value
Number of eyes, *n* (%)	18 (22.5)	62 (77.5)	
Sex, *n* (%)			
Male	11 (61.1)	43 (69.4)	0.572 *
Female	7 (38.9)	19 (30.6)	
Mean age, years	52 ± 15.3	60.9 ± 11.7	0.009 ^†^
Mean duration between symptom’s onset and surgery days	3.17 ± 1.4	9.5 ± 11.1	0.019 ^‡^
High myopia, *n* (%)	4 (22.2)	4 (6.45)	0.129 *
Macular status			
On, *n* (%)	6 (33.3)	20 (32.3)	1.000 *
Off, *n* (%)	12 (66.7)	42 (67.7)	
Lens status			
Phakic, *n* (%)	2 (11.1)	41 (66.1)	0 *
Pseudophakic, *n* (%)	16 (88.9)	21 (33.9)	
Type of surgery			
Pars plana vitrectomy, *n* (%)	16 (88.9)	51 (82.3)	0.721 *
Scleral buckling, *n* (%)	2 (11.1)	11 (17.7)	0.721 *
Number of ruptures, *n* (%)	1.83 ± 0.9	2.18 ± 1.2	0.363 ^‡^
Preoperative best-corrected visual acuity (BCVA) (logMAR), mean ± standard deviation (SD)	0.89 ± 0.83	1.2 ± 0.94	0.232 ^‡^
Subretinal fluid high 1 week postoperatively, µm	226.9 ± 260.9		
Systemic associated diseases			
Systemic hypertension, *n* (%)	5 (27)	11 (17.7)	0.338 *
Hypercholesterolemia, *n* (%)	3 (16)	9 (14.5)	1.000 *
Hypothyroidism, *n* (%)	1 (5)	2 (3.2)	0.539 *
Diabetes mellitus, *n* (%)	0	2 (3.2)	N/A

* = Fisher’s exact test; ^†^ = *t* test; ^‡^ Mann–Whitney U test. N/A= Not applicable.

**Table 2:** Risk factors for subretinal fluid (SRF) duration after surgery. Results of univariate analysis.

Risk Factor	Hazard Ratio	95.0% Confidence Interval	*p* Value
Age	−0.007	0.962–1.024	0.654
Sex	0.314	0.529–3.545	0.517
Laterality	0.036	0.410–2.620	0.939
Days between diagnosis and surgery	0.121	0.733–1.738	0.582
Number of ruptures	0.070	0.641–1.796	0.790
Localization of ruptures	−0.346	0.192–2.604	0.602
Preoperative best-corrected visual acuity (BCVA)	−0.731	0.228–1.014	0.054
BCVA at 6 months postoperatively	−0.409	0.003–173.822	0.885
SRF high at 1 week after surgery	−0.002	0.996–1.000	0.081
Lens status	0.209	0.272–5.572	0.787
Macula status	0.781	0.728–6.546	0.164
Type of surgery	1.087	0.647–13.591	0.162

**Table 3:** Risk factors for subretinal fluid (SRF) duration after surgery. Results of multivariate Cox regression analysis.

Risk Factor	Hazard Ratio	95.0% Confidence Interval	*p* Value
Preoperative best-corrected visual acuity (BCVA)	−1.110	0.094–1.153	0.082
SRF high at 1 week after surgery	−0.002	0.995–1.002	0.371
Macula status	−0.902	0.068–2.436	0.324
Type of surgery	0.299	0.105–17.280	0.818

## Data Availability

The original contributions presented in the study are included in the article, further inquiries can be directed to the corresponding author.
